# Supplementary material for: Improved performance and consistency of deep learning 3D liver segmentation with heterogeneous cancer stages in magnetic resonance imaging
Source: PLoS One. 2021 Dec 1;16(12):e0260630. doi: 10.1371/journal.pone.0260630 (PMC8635384; doi:10.1371/journal.pone.0260630)
Supplement: S1 Table — Magnetic resonance imaging parameters of the training, validation, and testing cohorts from 219 HCC patients included in this study. (DOCX) [file pone.0260630.s001.docx]

**Table S1. Magnetic resonance imaging parameters.** Magnetic resonance imaging parameters of the training, validation, and testing cohorts from 219 HCC patients included in this study.

| **Parameter** |  | **Overall** | **Training** | | | **Validation** | **Testing** |
| --- | --- | --- | --- | --- | --- | --- | --- |
|  |  |  | **Training Pool** | **EIS-Net** | **AS-Net** |  |  |
| n |  | 219 | 147 | 129 | 129 | 28 | 44 |
| Magnetic field strength (T) | 1.16 | 3  (1.4) | 2  (1.4) | 2  (1.6) | 2  (1.6) | 0 | 1  (2.3) |
|  | 1.5 | 143  (65.3) | 104  (70.7) | 90  (69.8) | 91  (70.5) | 18  (64.3) | 21  (47.7) |
|  | 3.0 | 73  (33.3) | 41  (27.9) | 37  (28.7) | 36  (27.9) | 10  (35.7) | 22  (50.0) |
| Manufacturer | GE Medical Systems | 47  (21.5) | 31  (21.1) | 29  (22.5) | 26  (20.2) | 7  (25.0) | 9  (20.5) |
|  | Hitachi Medical Corporation | 3  (1.4) | 2  (1.4) | 2  (1.6) | 2  (1.6) | 0 | 1  (2.3) |
|  | Philips | 2  (0.9) | 2  (1.4) | 2  (1.6) | 1  (0.8) | 0 | 0 |
|  | Siemens | 166  (75.8) | 111  (75.5) | 95  (73.6) | 99  (76.7) | 21  (75.0) | 34  (77.3) |
|  | Toshiba | 1  (0.5) | 1  (0.7) | 1  (0.8) | 1  (0.8) | 0 | 0 |
| Model name | Achieva | 1  (0.5) | 1  (0.7) | 1  (0.8) | 0 | 0 | 0 |
|  | Ingenia | 1  (0.5) | 1  (0.7) | 1  (0.8) | 1  (0.8) | 0 | 0 |
|  | Magnetom Aera | 38  (17.4) | 27  (18.4) | 24  (18.6) | 26  (20.2) | 2  (7.1) | 9  (20.5) |
|  | Magnetom Avanto | 40  (18.3) | 31  (21.1) | 26  (20.2) | 27  (20.9) | 6  (21.4) | 3  (6.8) |
|  | Magnetom Espree | 20  (9.1) | 16  (10.9) | 13  (10.1) | 16  (12.4) | 1  (3.6) | 3  (6.8) |
|  | Magnetom Skyra | 5  (2.3) | 4  (2.7) | 3  (2.3) | 4  (3.1) | 1  (3.6) | 0 |
|  | Magnetom Symphony | 3  (1.4) | 3  (2.0) | 3  (2.3) | 1  (0.8) | 0 | 0 |
|  | Magnetom Trio | 3  (1.4) | 0 | 0 | 0 | 1  (3.6) | 2  (4.5) |
|  | Magnetom Verio | 54  (24.7) | 29  (19.7) | 26  (20.2) | 24  (18.6) | 8  (28.6) | 17  (38.6) |
|  | Oasis | 3  (1.4) | 2  (1.4) | 2  (1.6) | 2  (1.6) | 0 | 1  (2.3) |
|  | Optima MR450w | 1  (0.5) | 1  (0.7) | 0 | 1  (0.8) | 0 | 0 |
|  | Signa Excite | 6  (2.7) | 4  (2.7) | 4  (3.1) | 2  (1.6) | 1  (3.6) | 1  (2.3) |
|  | Signa HDx | 22  (10.0) | 14  (9.5) | 14  (10.9) | 11  (8.5) | 3  (10.7) | 5  (11.4) |
|  | Signa HDxt | 2  (0.9) | 1  (0.7) | 1  (0.8) | 1  (0.8) | 0 | 1  (2.3) |
|  | not available | 20  (9.1) | 13  (8.8) | 11  (8.5) | 13  (10.1) | 5  (17.9) | 2  (4.5) |
| Contrast agent | Dotarem | 21  (9.6) | 13  (8.8) | 10  (7.8) | 13  (10.1) | 2  (7.1) | 6  (13.6) |
|  | Eovist | 8  (3.7) | 4  (2.7) | 4  (3.1) | 3  (2.3) | 2  (7.1) | 2  (4.5) |
|  | Gadavist | 107  (48.9) | 74  (50.3) | 67  (51.9) | 64  (49.6) | 12  (42.9) | 21  (47.7) |
|  | Magnevist | 36  (16.4) | 27  (18.4) | 25  (19.4) | 24  (18.6) | 2  (7.1) | 7  (15.9) |
|  | MultiHance | 5  (2.3) | 3  (2.0) | 1  (0.8) | 3  (2.3) | 2  (7.1) | 0 |
|  | Omniscan | 3  (1.4) | 2  (1.4) | 2  (1.6) | 2  (1.6) | 1  (3.6) | 0 |
|  | OpiMARK | 2  (0.9) | 2  (1.4) | 2  (1.6) | 2  (1.6) | 0 | 0 |
|  | ProHance | 2  (0.9) | 1  (0.7) | 0 | 1  (0.8) | 1  (3.6) | 0 |
|  | not available | 35  (16.0) | 21  (14.3) | 18  (14.0) | 17  (13.2) | 6  (21.4) | 8  (18.2) |
| Contrast bolus volume (mL), median [Q1,Q3] |  | 10.1  [8.0,  16.0] | 10.4  [8.2,  16.0] | 10.2  [7.8,  16.0] | 10.6  [8.0,  16.0] | 10.2  [8.0,  17.5] | 10.0  [8.3,  16.0] |
| Repetition time (ms), mean (SD) |  | 4.3  (0.8) | 4.3  (0.7) | 4.3  (0.8) | 4.3  (0.8) | 4.1  (0.6) | 4.4  (0.9) |
| Imaging frequency (MHz), median [Q1,Q3] |  | 63.8  [63.6,  123.1] | 63.7  [63.6,  123.1] | 63.7  [63.6,  123.1] | 63.7  [63.6,  123.1] | 63.8  [63.7,  123.2] | 93.5  [63.6,  123.2] |
| Slice thickness (mm), median [Q1,Q3] |  | 3.0  [3.0,4.0] | 3.0  [3.0,4.0] | 3.0  [3.0,4.0] | 3.0  [3.0,4.0] | 3.0  [3.0,4.1] | 3.0  [3.0,3.5] |
| Spacing between slices (mm), mean (SD) |  | 2.4  (0.6) | 2.4  (0.6) | 2.4  (0.5) | 2.5  (0.6) | 2.2  (0.2) | 2.6  (0.7) |
| Specific absorption rate (W/kg), median [Q1,Q3] |  | 0.3  [0.2,0.4] | 0.3  [0.1,0.4] | 0.3  [0.2,0.4] | 0.3  [0.1,0.4] | 0.4  [0.2,0.5] | 0.3  [0.2,0.4] |
| Bandwidth (Hz), median [Q1,Q3] |  | 445.0  [345.0,  446.0] | 425.0  [345.0,  446.0] | 423.0  [334.0,  446.0] | 434.0  [345.0,  446.0] | 445.0  [309.1,  446.0] | 445.0  [345.0,  446.0] |
| Phase FOV (%), mean (SD) |  | 83.0  (8.6) | 83.1  (8.9) | 83.3  (8.7) | 83.1  (9.0) | 83.7  (8.7) | 82.2  (7.6) |

Numbers in parentheses are percentages if not indicated otherwise. EIS-Net = Early-Intermediate-Stage-Net, AS-Net = All-Stage-Net
