# Supplementary material for: Improved performance and consistency of deep learning 3D liver segmentation with heterogeneous cancer stages in magnetic resonance imaging
Source: PLoS One. 2021 Dec 1;16(12):e0260630. doi: 10.1371/journal.pone.0260630 (PMC8635384; doi:10.1371/journal.pone.0260630)
Supplement: S2 Table — Dice Similarity Coefficient (DSC) results for the Early-Intermediate-Stage-Net (EIS-Net) and All-Stage-Net (AS-Net) compared against the experts’ manual segmentations. (DOCX) [file pone.0260630.s002.docx]

**Table S2. Dice Similarity Coefficient (DSC) results.** Dice Similarity Coefficient (DSC) results for the Early-Intermediate-Stage-Net (EIS-Net) and All-Stage-Net (AS-Net) compared against the experts’ manual segmentations.

|  |  | EIS-Net | | | AS-Net | | |  |
| --- | --- | --- | --- | --- | --- | --- | --- | --- |
|  | Count | Mean | SD | Median | Mean | SD | Median | p-Value |
| Child-Pugh Score  A | 30 | 0.946 | 0.036 | 0.958 | 0.953 | 0.020 | 0.960 | 0.010 * |
| B | 6 | 0.944 | 0.015 | 0.945 | 0.944 | 0.016 | 0.948 | 0.688 |
| C | 8 | 0.950 | 0.023 | 0.962 | 0.962 | 0.009 | 0.965 | 0.008 * |
| Disease involving % of  hepatic Parenchyma  <50% | 36 | 0.943 | 0.034 | 0.955 | 0.951 | 0.019 | 0.958 | 0.005 * |
| ≥50% | 8 | 0.961 | 0.012 | 0.961 | 0.965 | 0.011 | 0.967 | 0.023 * |
| Ascites on imaging  absent | 35 | 0.945 | 0.034 | 0.957 | 0.953 | 0.020 | 0.961 | 0.013 * |
| present | 9 | 0.950 | 0.018 | 0.952 | 0.957 | 0.010 | 0.952 | 0.004 * |
| Cumulative tumor diameter  <3cm | 18 | 0.940 | 0.043 | 0.957 | 0.952 | 0.018 | 0.958 | 0.090 |
| ≥3cm | 26 | 0.950 | 0.021 | 0.956 | 0.955 | 0.019 | 0.962 | 0.002 * |

* indicates statistically significant differences between EIS-Net and AS-Net.
