# Supplementary material for: Improved performance and consistency of deep learning 3D liver segmentation with heterogeneous cancer stages in magnetic resonance imaging
Source: PLoS One. 2021 Dec 1;16(12):e0260630. doi: 10.1371/journal.pone.0260630 (PMC8635384; doi:10.1371/journal.pone.0260630)
Supplement: S3 Table — Modified Hausdorff Distance (MHD) (in voxels) results for the Early-Intermediate-Stage-Net (EIS-Net) and All-Stage-Net (AS-Net) compared against the experts’ manual segmentations. (DOCX) [file pone.0260630.s003.docx]

**Table S3. Modified Hausdorff Distance (MHD) results**. Modified Hausdorff Distance (MHD) (in voxels) results for the Early-Intermediate-Stage-Net (EIS-Net) and All-Stage-Net (AS-Net) compared against the experts’ manual segmentations.

|  |  | EIS-Net | | | AS-Net | | |  |
| --- | --- | --- | --- | --- | --- | --- | --- | --- |
|  | Count | Mean | SD | Median | Mean | SD | Median | p-Value |
| Child-Pugh Score  A | 30 | 5.343 | 8.343 | 2.236 | 3.647 | 4.588 | 2.236 | 0.044 * |
| B | 6 | 4.467 | 4.007 | 3.146 | 4.514 | 3.626 | 3.371 | 0.500 |
| C | 8 | 8.582 | 12.921 | 2.532 | 2.191 | 0.631 | 2.236 | 0.028 * |
| Disease involving % of  hepatic parenchyma  <50% | 36 | 6.460 | 9.640 | 2.236 | 3.741 | 4.411 | 2.236 | 0.007 * |
| ≥50% | 8 | 2.899 | 1.240 | 2.850 | 2.418 | 1.015 | 2.236 | 0.225 |
| Ascites on imaging  absent | 35 | 6.153 | 9.674 | 2.236 | 3.643 | 4.501 | 2.236 | 0.014 * |
| present | 9 | 4.488 | 4.253 | 3.464 | 2.943 | 0.951 | 2.449 | 0.093 |
| Cumulative tumor diameter  <3cm | 18 | 5.665 | 9.594 | 2.236 | 3.153 | 2.798 | 2.236 | 0.385 |
| ≥3cm | 26 | 5.915 | 8.441 | 2.828 | 3.741 | 4.745 | 2.236 | 0.003 * |

* indicates statistically significant differences between EIS-Net and AS-Net.
