# Supplementary material for: Improved performance and consistency of deep learning 3D liver segmentation with heterogeneous cancer stages in magnetic resonance imaging
Source: PLoS One. 2021 Dec 1;16(12):e0260630. doi: 10.1371/journal.pone.0260630 (PMC8635384; doi:10.1371/journal.pone.0260630)
Supplement: S4 Table — Mean Absolute Distance (MAD) (in voxels) results for the Early-Intermediate-Stage-Net (EIS-Net) and All-Stage-Net (AS-Net) compared against the experts’ manual segmentations. (DOCX) [file pone.0260630.s004.docx]

**Table S4. Mean Absolute Distance (MAD) results.** Mean Absolute Distance (MAD) (in voxels) results for the Early-Intermediate-Stage-Net (EIS-Net) and All-Stage-Net (AS-Net) compared against the experts’ manual segmentations.

|  |  | EIS-Net | | | AS-Net | | |  |
| --- | --- | --- | --- | --- | --- | --- | --- | --- |
|  | Count | Mean | SD | Median | Mean | SD | Median | p-Value |
| Child-Pugh Score  A | 30 | 1.206 | 1.975 | 0.699 | 0.794 | 0.433 | 0.662 | 0.057 |
| B | 6 | 0.749 | 0.158 | 0.716 | 0.729 | 0.099 | 0.752 | 1.000 |
| C | 8 | 1.753 | 2.347 | 0.657 | 0.600 | 0.152 | 0.575 | 0.008 * |
| Disease involving % of  hepatic parenchyma  <50% | 36 | 1.347 | 2.090 | 0.698 | 0.783 | 0.398 | 0.683 | 0.046 * |
| ≥50% | 8 | 0.777 | 0.266 | 0.760 | 0.601 | 0.134 | 0.598 | 0.039 * |
| Ascites on imaging  absent | 35 | 1.318 | 2.103 | 0.703 | 0.776 | 0.405 | 0.666 | 0.053 |
| present | 9 | 0.953 | 0.717 | 0.646 | 0.649 | 0.159 | 0.610 | 0.012 * |
| Cumulative tumor diameter  <3cm | 18 | 1.502 | 2.549 | 0.655 | 0.822 | 0.515 | 0.643 | 0.142 |
| ≥3cm | 26 | 1.064 | 1.311 | 0.737 | 0.701 | 0.221 | 0.626 | 0.018 * |

* indicates statistically significant differences between EIS-Net and AS-Net.
