# Supplementary material for: Improved performance and consistency of deep learning 3D liver segmentation with heterogeneous cancer stages in magnetic resonance imaging
Source: PLoS One. 2021 Dec 1;16(12):e0260630. doi: 10.1371/journal.pone.0260630 (PMC8635384; doi:10.1371/journal.pone.0260630)
Supplement: S1 File — (DOCX) [file pone.0260630.s005.docx]

Deep learning model architecture

An adopted 3D U-net architecture was used and implemented in the open-source Medical Open Network for AI (MONAI) (v0.3.0) framework [48]. This network was parameterized to have encoding blocks with the following components: a convolution layer of stride 2 to downsample the data followed by a residual unit consisting of two units of convolution, normalization (Batch Norm), dropout, and activation (PReLU) repeated in succession. The parameterization uses four encoding blocks with 16, 32, 64, and 128 features, respectively. The decoding blocks take the form: a convolution transpose layer to upsample the data followed by Batch Norm, dropout, and PReLU layers and then a residual unit consisting of convolution, Batch Norm, dropout, and PReLU. All convolution operations use 3x3x3 kernels, and the convolution transpose operations use 2x2x2 kernels with stride size 2. The final segmentation convolution layer uses a 1x1x1 kernel with stride 1 and has 2 features. In total, the model contains 1,187,921 trainable parameters.

Model weights were initialized using the default PyTorch method to have random weights uniformly sampled between [-k,+k] where $k=\sqrt{1/kernel size}$.

No data augmentation was used.

To remove segmentation artifacts, all results were post-processed to keep only the largest connected component (the estimated liver mask) in the image.

Full network details can be found in the source code repository (<https://github.com/OnofreyLab/liver-segm>).

Segmentation performance metrics

To quantitatively assess segmentation performance, the following measures were calculated: Dice Similarity Coefficient (DSC), Modified Hausdorff Distance (MHD), and Mean Absolute Distance (MAD).

1. DSC measures the overlap between the predicted liver segmentation and the manual ground-truth:

$$DSC(A,B)=\frac{2A\cap B}{A+B}$$

Where A and B are the segmentation predictions and ground-truth images, respectively.

To calculate MHD and MAD, the liver segmentation surfaces A and B were extracted.

1. Hausdorff Distance (HD) is calculated as:

$$HD(A,B)=max\left\{ \max_{a\in A} d(a,B),\left. \max_{b\in B} d(b,A) \right\} \right.$$

where $d(a,B)= {min}_{b \in B}\left\| b-a \right\|^{2}$.

And the MHD weas calculated, which is less sensitive to outliers, by using the 95th percentile of *d*(a,B) instead of using the maximum value as in HD.

1. MAD was calculated as:

$$MAD(A,B)=\frac{1}{2}\left\{ \frac{1}{N_{A}}\sum_{a\in A} d(a,B)+\frac{1}{N_{B}}\sum_{b\in B} d(b,A) \right\}$$
