# Supplementary material for: Improved performance and consistency of deep learning 3D liver segmentation with heterogeneous cancer stages in magnetic resonance imaging
Source: PLoS One. 2021 Dec 1;16(12):e0260630. doi: 10.1371/journal.pone.0260630 (PMC8635384; doi:10.1371/journal.pone.0260630)
Supplement: Attachment — Submitted filename: PONE-D-21-18482_response_final.docx [file pone.0260630.s006.docx]

We thank the reviewers and editor for their time spent reading our manuscript and for their thoughtful comments. We have prepared detailed responses to each comment (*in italics*) and have incorporated changes into our revised manuscript using Track Changes (with changes highlighted in blue text). We begin with a summary of major changes made to this revised manuscript.

**Summary of Major Changes**

The revised version of our manuscript includes the following major changes:

1. In response to Reviewer 2, we have revised the manuscript to highlight the contributions and significance:
   a. We have elaborated on the challenges of distributional shift in machine learning in both the Introduction and the Discussion. Our results demonstrate the problem of distributional shift in liver segmentation and how training across the distribution clinical liver cancer imaging data can make model performance more robust by reducing this shift.
   b. We have provided additional references to recent liver segmentation approaches to make our manuscript more comprehensive in both the Introduction and the Discussion sections.
2. Addressed specific journal requirements for submission that include:
   a. Ensured that the manuscript meets PLOS ONE’s style requirements.
   b. Removal of all funding-related text from the manuscript’s Acknowledgements section.
   c. Updated the Data Availability statement.
   d. Moved the Ethics Statement to appear within the Methods section.
   e. All figures are available under the CC BY 4.0 license.

Reviewers' Comments:

**Reviewer 1: Comments to the Author and Responses**

Reviewer #1: Well written article. The presented works are reproducible, proposed methodology writing is clear and easy to follow. The writing content demonstrated a high technically sound piece of research works. Presented outcome is convincing with enough simulated samples.

*We thank the reviewer for their time and positive assessment or our manuscript’s reproducibility, technical value, and clear writing. We have revised the manuscript to address comments from Reviewer 2 and to match journal requirements without sacrificing the meritorious contributions noted by Reviewer 1.*

**Reviewer 2: Comments to the Author and Responses**

Reviewer #2: In this research, authors wish to assess the ability of state-of-the-art deep learning 3D liver segmentation algorithms to generalize across different BCLC liver cancer stages. The research is interesting, however, the proposed methodology existed in the literature and thus the novelty of the research is not sufficient to be published in PLOS ONE. Furthermore, the discussions and also the analysis of the manuscript need to be further improve in order to be published in PLOS ONE. So I suggest reject the article and giving the author a chance to submit after doing additional research to improve the contributions of the research work. Authors may also consider the following comments for the revision work:

*We thank the reviewer for their constructive feedback and provide detailed responses to each comment below.*

1. The writing of the manuscript need to be further improve. The sections and sub sections need to be change. E.g. there should be sections like Literature review, Methodology, Results and Discussions etc. There are some crucial sections missing in the manuscript. The flow is a bit difficult to follow.

*We have revised the format by removing section and sub-section numbering and ensured proper heading formats. Our manuscript includes the required section headings specified by the PLOS ONE submission guidelines (*[*https://journals.plos.org/plosone/s/submission-guidelines#loc-manuscript-organization*](https://journals.plos.org/plosone/s/submission-guidelines#loc-manuscript-organization)*): Title page; Abstract; Introduction; Materials and Methods; Results; Discussion; Conclusions; and References.*

*Within this format, we have incorporated all relevant literature review into the top level Introduction section.*

2. The format of the references and citations need to be improve. For the literature review, authors should refer to more recent research, i.e. year 2019-2020. Currently there is only 1 2020 reference which is not sufficient.

*We have formatted all references according to the Reference style Guidelines* *(*[*https://journals.plos.org/plosone/s/submission-guidelines#loc-manuscript-organization*](https://journals.plos.org/plosone/s/submission-guidelines#loc-manuscript-organization)*) to use the “Vancouver” bibliography style.*

*We have also included 10 new citations to review current work in the field of liver segmentation. These have been added to the Introduction and Discussion sections where appropriate.*

3. Authors need to identify the research gap and highlight the contribution(s) of the research work in the manuscript to shows the novelty of the research.

*The main research gap in applying deep learning models to clinical data involves a shift in the distribution between model training data and testing data. This problem is particularly challenging in medical imaging applications where limited data samples are available, and this has a negative impact on translational impact of these algorithms when applied to clinical data. In the real world, clinical data is heterogeneous, and, in this manuscript, we demonstrate that data from the full distribution of cancer imaging data is required to create a model that is robust to these distributional shifts. We have added text to the Introduction to discuss the significance of distributional shifts and added text to the Discussion to highlight the contributions of this manuscript.*

4. Authors should compare the proposed techniques with the state-of-arts to further prove the contribution(s)/novelty of the research work.

*The contributions of this work are to demonstrate and to quantify the effects of training a deep learning segmentation model with data that is not representative of the heterogeneous distribution of data observed in real world clinical practice. Our results demonstrate the importance of training with imaging data from the full distribution of clinical images in liver cancer using a state-of-the-art (SOTA) deep learning segmentation architecture. The goal of this work was not to develop a novel segmentation approach, but instead see how the SOTA approach is susceptible to problems of distributional shifts between training and testing data.*

*Furthermore, deep learning segmentation networks based on the U-net architecture remain the SOTA in medical image segmentation. We have applied a SOTA U-net model to the problem of liver segmentation and demonstrate results that are either concordant or superior to other published literature while using a challenging test data that includes samples from early, intermediate, and advanced stage liver cancer.*

5. More scientific reasoning should be added in the experimental results' explanations.

*We have elaborated on the problems of distributional shift in machine learning in the Introduction and the Discussion. Distributional shift, or dataset shift, is a major problem in machine learning, and is particularly challenging for deep learning applications that feature non-linear and high-dimensional model complexity. We have provided references from the literature to highlight this problem and its significance to medical image analysis. We have revised our manuscript to put our experimental results within the context of distributional shift.*

6. The format of the tables need to be improved. The current one is a bit difficult to read.

*We have reformatted all tables according to the PLOS Manuscript Body Formatting Guidelines (*[*https://journals.plos.org/plosone/s/file?id=9cba/PLOS%20Manuscript%20Body%20Formatting%20Guidelines.pdf*](https://journals.plos.org/plosone/s/file?id=9cba/PLOS%20Manuscript%20Body%20Formatting%20Guidelines.pdf)*).*

**Journal Requirements:**

When submitting your revision, we need you to address these additional requirements.

1. Please ensure that your manuscript meets PLOS ONE's style requirements, including those for file naming. The PLOS ONE style templates can be found at 
<https://journals.plos.org/plosone/s/file?id=wjVg/PLOSOne_formatting_sample_main_body.pdf> and 
<https://journals.plos.org/plosone/s/file?id=ba62/PLOSOne_formatting_sample_title_authors_affiliations.pdf>

*We have ensured that our manuscript satisfies the PLOS ONE style requirements.*

2. Thank you for stating the following in the Acknowledgments Section of your manuscript: 
[Research reported in this publication was supported by the National Institute of Diabetes and Digestive and Kidney Diseases of the National Institutes of Health under Award Number P30 KD034989 and the National Institutes of Health Grant Award Number DDRCC DK034989-36 for the Clinical Translational Core of the Yale Liver Center. The content is solely the responsibility of the authors and does not necessarily represent the official views of the Nation Institutes of Health. M.G. was supported by a travel grant by the Rolf W. Günther Foundation for Radiological Sciences for travel to Yale University.]
We note that you have provided funding information that is not currently declared in your Funding Statement. However, funding information should not appear in the Acknowledgments section or other areas of your manuscript. We will only publish funding information present in the Funding Statement section of the online submission form. 
Please remove any funding-related text from the manuscript and let us know how you would like to update your Funding Statement. Currently, your Funding Statement reads as follows: 
 [J.O. was supported by the National Institute of Diabetes and Digestive and Kidney Diseases of the National Institutes of Health under Award Number P30 KD034989 and M.S. the National Institutes of Health Grant Award Number DDRCC DK034989-36 for the Clinical Translational Core of the Yale Liver Center. The content is solely the responsibility of the authors and does not necessarily represent the official views of the Nation Institutes of Health. M.G. was supported by a travel grant by the Rolf W. Günther Foundation for Radiological Sciences for travel to Yale University.The funders had no role in study design, data collection and analysis, decision to publish, or preparation of the manuscript.]

Please include your amended statements within your cover letter; we will change the online submission form on your behalf.

*We have removed all funding-related text from the manuscript’s Acknowledgements section.*

3. your Data Availability statement, you have not specified where the minimal data set underlying the results described in your manuscript can be found. PLOS defines a study's minimal data set as the underlying data used to reach the conclusions drawn in the manuscript and any additional data required to replicate the reported study findings in their entirety. All PLOS journals require that the minimal data set be made fully available. For more information about our data policy, please see <http://journals.plos.org/plosone/s/data-availability>.

Upon re-submitting your revised manuscript, please upload your study’s minimal underlying data set as either Supporting Information files or to a stable, public repository and include the relevant URLs, DOIs, or accession numbers within your revised cover letter. For a list of acceptable repositories, please see <http://journals.plos.org/plosone/s/data-availability#loc-recommended-repositories>. Any potentially identifying patient information must be fully anonymized.

Important: If there are ethical or legal restrictions to sharing your data publicly, please explain these restrictions in detail. Please see our guidelines for more information on what we consider unacceptable restrictions to publicly sharing data: <http://journals.plos.org/plosone/s/data-availability#loc-unacceptable-data-access-restrictions>. Note that it is not acceptable for the authors to be the sole named individuals responsible for ensuring data access.

We will update your Data Availability statement to reflect the information you provide in your cover letter.

*Imaging data files cannot be shared due to patient privacy restrictions at Yale. We are making our trained model fully available on GitHub.*

4. Please note that in order to use the direct billing option the corresponding author must be affiliated with the chosen institute. Please either amend your manuscript to change the affiliation or corresponding author, or email us at [plosone@plos.org](mailto:plosone@plos.org) with a request to remove this option.

*The corresponding author John Onofrey is affiliated with Yale University. Yale University is an Institutional Partner with all PLOS journals, and will cover the cost of publishing if accepted for publication.*

5. Your ethics statement should only appear in the Methods section of your manuscript. If your ethics statement is written in any section besides the Methods, please move it to the Methods section and delete it from any other section. Please ensure that your ethics statement is included in your manuscript, as the ethics statement entered into the online submission form will not be published alongside your manuscript.

*We have moved the Ethics Statement to appear within the Methods section as requested.*

6. We note that Figures 1, 3 and 4 in your submission contain copyrighted images. All PLOS content is published under the Creative Commons Attribution License (CC BY 4.0), which means that the manuscript, images, and Supporting Information files will be freely available online, and any third party is permitted to access, download, copy, distribute, and use these materials in any way, even commercially, with proper attribution. For more information, see our copyright guidelines: <http://journals.plos.org/plosone/s/licenses-and-copyright>.
 
We require you to either (1) present written permission from the copyright holder to publish these figures specifically under the CC BY 4.0 license, or (2) remove the figures from your submission:
 
a) You may seek permission from the original copyright holder of Figures 1, 3 and 4 to publish the content specifically under the CC BY 4.0 license. 
 
We recommend that you contact the original copyright holder with the Content Permission Form (<http://journals.plos.org/plosone/s/file?id=7c09/content-permission-form.pdf>) and the following text:
“I request permission for the open-access journal PLOS ONE to publish XXX under the Creative Commons Attribution License (CCAL) CC BY 4.0 (<http://creativecommons.org/licenses/by/4.0/>). Please be aware that this license allows unrestricted use and distribution, even commercially, by third parties. Please reply and provide explicit written permission to publish XXX under a CC BY license and complete the attached form.”
 
Please upload the completed Content Permission Form or other proof of granted permissions as an "Other" file with your submission. 
 
In the figure caption of the copyrighted figure, please include the following text: “Reprinted from [ref] under a CC BY license, with permission from [name of publisher], original copyright [original copyright year].”
 
b) If you are unable to obtain permission from the original copyright holder to publish these figures under the CC BY 4.0 license or if the copyright holder’s requirements are incompatible with the CC BY 4.0 license, please either i) remove the figure or ii) supply a replacement figure that complies with the CC BY 4.0 license. Please check copyright information on all replacement figures and update the figure caption with source information. If applicable, please specify in the figure caption text when a figure is similar but not identical to the original image and is therefore for illustrative purposes only.

*The original copyright holder (Moritz Gross) has granted permission for copyright transfer for all images to be published under the CC BY 4.0 license. We have uploaded the corresponding copyright transfer information as a pdf with the submission.*
