# Supplementary material for: Improved performance and consistency of deep learning 3D liver segmentation with heterogeneous cancer stages in magnetic resonance imaging
Source: PLoS One. 2021 Dec 1;16(12):e0260630. doi: 10.1371/journal.pone.0260630 (PMC8635384; doi:10.1371/journal.pone.0260630)
Supplement: Attachment — Submitted filename: PONE-D-21-18482_R2_response_final.docx [file pone.0260630.s007.docx]

We thank the reviewers and editor for their time spent reading our manuscript and for their thoughtful comments. We have prepared detailed responses to each comment (*in italics*) and have incorporated changes into our revised manuscript using Track Changes (with changes highlighted in blue text).

**Reviewers' Comments:**

Please improve each caption of figure and title for table to assure that take-home message was incorporated into the caption or title so that the figure can be standalone without requiring readers to read the text to understand the gist of the figure/table. In other words, highlight the findings of the figure/table using caption/title to facilitate reading.

*We have changed the figure captions and table titles so that they contain and highlight the main message and can be understood without reading the text.*

Besides, please assure that the Github link can be accessed,.

*The Github link can now be accessed through the following link: https://github.com/OnofreyLab/liver-segm*

Lastly, you are advised to revise your conclusion based on the result acquired from the experiment; reiterate and specify the critical findings based on experimental result and then only explain the implication of these findings to the field of study.

*We have revised our conclusion and made it more specific to our study and field of research.*
